# Surviving murine experimental sepsis affects the function and morphology of the inner ear

**DOI:** 10.1242/bio.024588

**Published:** 2017-06-15

**Authors:** Natalie Fischer, Nina Maria Mathonia, Georges Hoellerich, Julian Veser, Leyla Pinggera, Daniel Dejaco, Rudolf Glueckert, Anneliese Schrott-Fischer, Peter Lackner, Herbert Riechelmann, Joachim Schmutzhard

**Affiliations:** 1 Department of Otorhinolaryngology, Medical University of Innsbruck, 6020 Innsbruck, Austria; 2 Department of Neurology, Medical University of Innsbruck, 6020 Innsbruck, Austria

**Keywords:** Sepsis, Hearing loss, Inner ear, Inflammation, Apoptosis

## Abstract

Severe sepsis is known to result in various neurological long-term deficits in human. Recently, a link between severe, lethal sepsis and significant hearing loss with correlating histomorphological inner ear changes in mice (C57BL/6) was observed. However, if similar observations can be made in severe, non-lethal sepsis in mice is unclear. This study evaluates mice after severe, non-lethal sepsis for analogue functional and histomorphological alterations of the inner ear.

A total of 63 C57BL/6 mice were included in the study. All underwent an initial hearing test with auditory brainstem response on day 1. In 35 mice sepsis was induced by cecal ligation and puncture (CLP), in 15 sham surgery was performed and 13 served as healthy control. A second hearing test was performed on day 7. All mice were sacrificed afterwards for further histomorphological evaluation of the inner ears. Immunohistochemical analysis with apoptotic markers Cleaved-caspase 3, BAX and BCL-2 were performed to identify structural inner ear damage.

Of all CLP mice, 21/35 (60.0%) died due to the induced sepsis. Of the surviving CLP mice, 14/35 (40.0%), post-treatment hearing thresholds differed significantly from the sham and control mice (*P*<0.001). Positive immunostaining at different inner ear structures, like the spiral ligament or the supporting cells could be observed. The percentage of the immunostained positive area in the spiral ligament significantly correlated with the grade of hearing loss for BAX (*P*=0.027) and Cleaved-caspase 3 (*P*=0.024) but not for BCL 2 (*P*>0.05).

The present data suggests that severe, non-lethal sepsis in mice results in significantly elevated hearing thresholds. A positive labelling for the pro-apoptotic markers BAX and Cleaved-caspase 3 suggested the induction of apoptosis in inner ear.

## Abstract (precis)

**Summary:** This study shows that a mild sepsis leads to a significant hearing impairment due to apoptosis of different inner ear structures.

## INTRODUCTION

Sepsis is a severe disease, which can result in multiple organ failure and death. Survivors suffer long-term sequelae. The responsible mechanisms for the chronic consequences of severe acute inflammation are not well understood (Benjamim et al., 2004).

Hearing impairment is a serious complication associated with critical illness and goes along with a significant impact on the social life, causing feelings of loneliness, isolation and depression. Due to these implications hearing loss is one of the six most frequent diseases which impair the quality of life extensively (Li et al., 2014; Mick et al., 2014).

The causes of hearing loss in critical ill patients in the intensive care unit (ICU) range from mechanical or accidental trauma, administration of ototoxic medications, local or systemic infections, vascular and hematologic disorders, autoimmune diseases to environmental noise (Halpern et al., 1999). Severe sepsis in humans is frequently treated with ototoxic medications, like antibiotics or diuretics etc. Therefore a differentiation of the fundamental cause of the resulting hearing impairment has not been possible so far.

One possible influential factor resulting in hearing loss due to severe acute inflammation, like severe sepsis, has been reported by Schmutzhard and colleagues in a different setting. In an experimental murine malaria model, a significant hearing loss was observed. Furthermore, the histopathologic evaluation revealed apoptosis of fibrocytes of the spiral ligament. Malaria typical changes were not identified. The authors discussed systemic inflammatory response (SIRS) as possible cause for the observed hearing loss (Schmutzhard et al., 2012). Consequently, a comparable investigation was performed utilising an experimental murine sepsis model. Instead of infecting mice with malaria, sepsis was induced in C57BL/6 mice via cecal ligation and puncture (CPL). In this setting, a significant increase in auditory brainstem response (ABR) thresholds was observed. Moreover, an induction of apoptosis in the supporting cells of the organ of Corti and an excitotoxicity at the basal pole of the inner hair cells in histopathological evaluation was reported. Thus, SIRS as common feature of malaria and sepsis was discussed as possible cause for hearing loss by the authors (Schmutzhard et al., 2013).

These observations were supported by two other experimental studies. Moon and colleagues observed a release of tumour necrosis factor-α from spiral ligament fibrocytes, if exposed to otitis media pathogens (Moon et al., 2006). Furthermore, Uchida and co-authors reported a possible involvement of the signalling cascades of tumour necrosis factor-α (TNF-α) in hearing impairment (Uchida et al., 2014). TNF-α and TNF-α receptor interactions were discussed as key elements in the pathogenesis of SIRS and apoptosis (Schulte et al., 2013).

To detect apoptosis different markers were established the downregulation of the anti-apoptotic pathways can be demonstrated with decreased Bcl-2 labelling, which needs to be compared with the pro-apoptotic antagonist, the Bax protein. A Bax/Bcl-2 ratio favouring Bax expression with additional downstream Cleaved-caspase 3 (CC-3) upregulation is a solid sign for induced apoptosis (Salakou et al., 2007; Yang and Korsmeyer, 1996).

The link of hearing loss and severe, lethal experimental sepsis appears established. However, no such link has been made in a non-lethal experimental murine sepsis model yet. This study evaluates whether hearing impairment occurs in a non-lethal experimental sepsis and implies pathologic alterations of inner ear after survived disease.

## RESULTS

63 two-month-old C57BL/6 mice (Charles River, Sulzfeld, Germany) were eligible for the study after the primary ABR measurement. Sepsis was induced in 35 mice with cecal ligation puncture, 15 mice underwent sham surgery (laparotomy, no ligation, no puncture) and 13 mice served as control (identical housing and treatment, but no intervention). 21/35 (60.0%) of the CLP mice died due to the induced sepsis.

### Evaluation of hearing performance

**Fig. 1.**
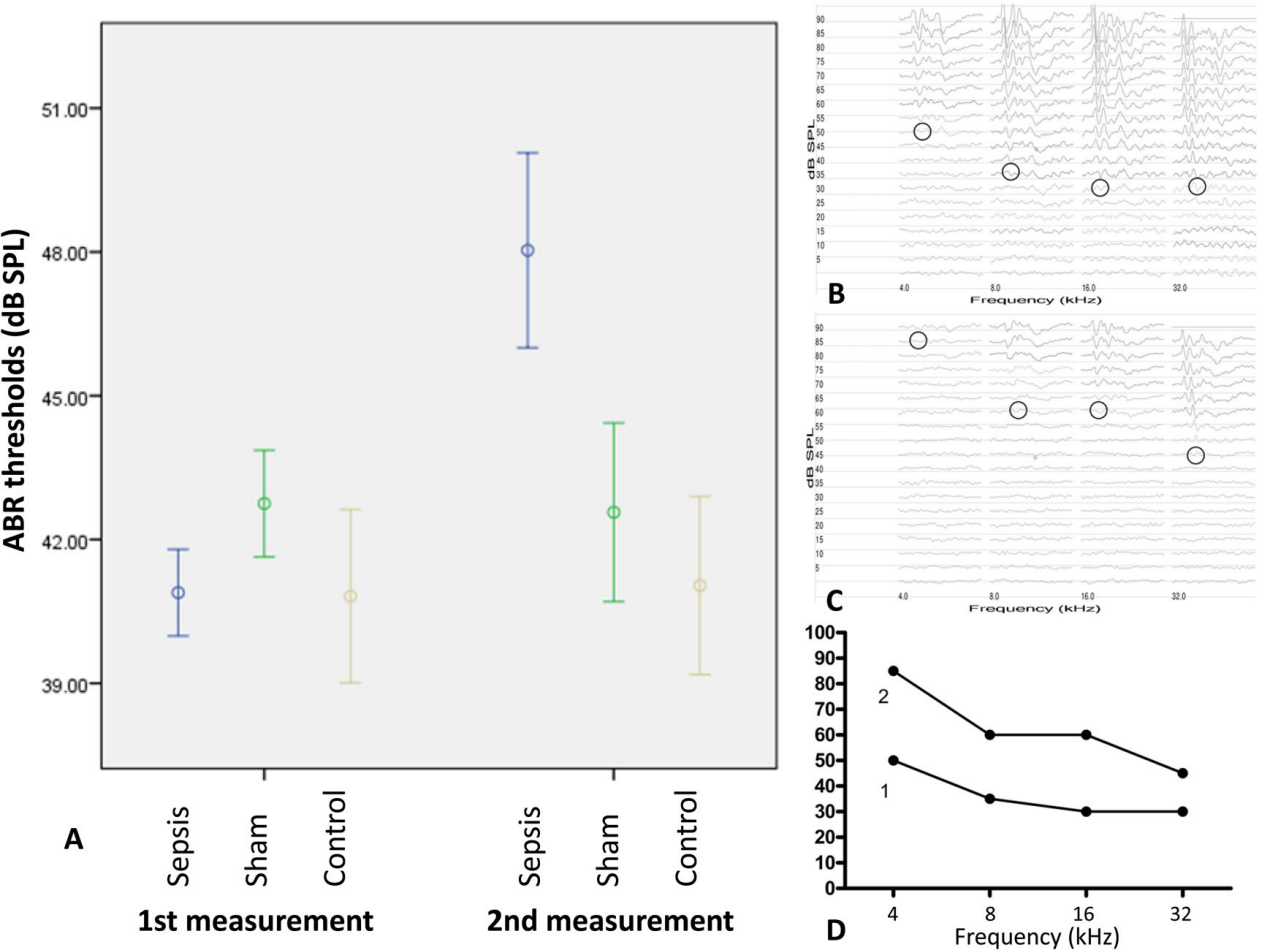
**ABR thresholds at the first measurement (day one) and the second measurement (day seven).** (A) In sepsis mice an average hearing loss of 7.1 dB is shown, while no change of the ABR threshold in the control and sham group can be seen (*P*=0.001). Error bars indicate standard error of mean. (B-D) Representative ABR results of a mouse suffering from sepsis. The initial ABR thresholds (B) increased clearly at the second measurement which was performed at day seven (C). (D) Comparison of the ABR thresholds at the first and the second measurement.

At day one an initial objective hearing test was performed with auditory evoked brainstem responses. The mean pure tone average in the sepsis group was 40.9 dB (SD5.4), in the sham group 42.8 dB (SD4.3) and in the control group 40.8 dB (SD6.5) (Fig. 1A).

At day 7 the mean hearing thresholds of surviving mice in the sepsis group was 48.0 dB (SD7.6), in the sham group 42.6 dB (SD6.5) and in the control group 41.0 dB (SD6.7) (Fig. 1B).

The pre- and post-treatment average hearing threshold and the mean hearing loss in sepsis mice at different frequencies of 4, 8, 16 and 32 KHz were shown in Table 1. Fig. 1B-D shows a representative ABR threshold of a mouse before and after survived sepsis (Fig. 1B-D).

**Table 1.**
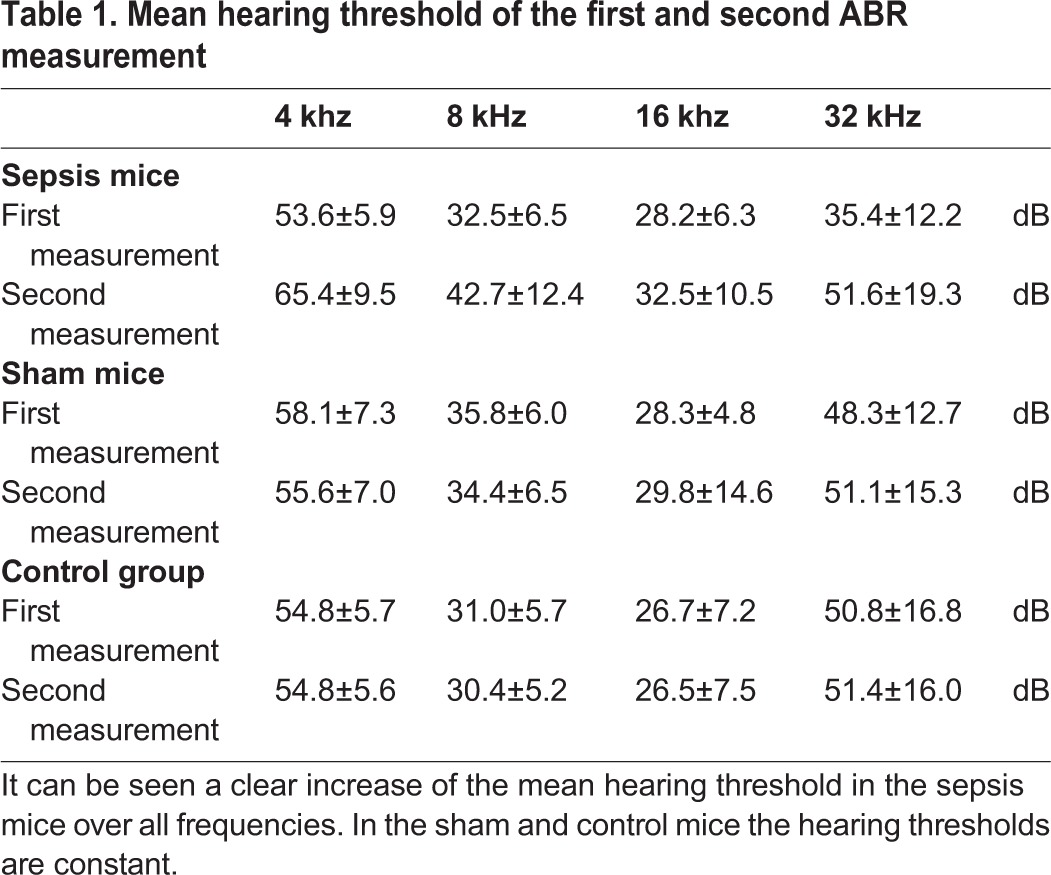
**Mean hearing threshold of the first and second ABR measurement**

Post-treatment thresholds of the surviving sepsis mice differed significantly from the sham and control group (*P*<0.001). While in the sepsis group an average hearing loss of 9.8 dB was found, no change of hearing thresholds could be seen in the sham and control mice (Fig. 1A).

### Immunohistochemical analysis

For the immunohistochemical analysis five cochleae with a high hearing loss, three mice with low hearing loss, four mice of the sham group and one healthy control were randomly included.

With light microscopy no morphological pathological findings in the organ of Corti, the stria vascularis and the spiral ganglion neurons could be found.

### Cleaved-caspase 3

**Fig. 2.**
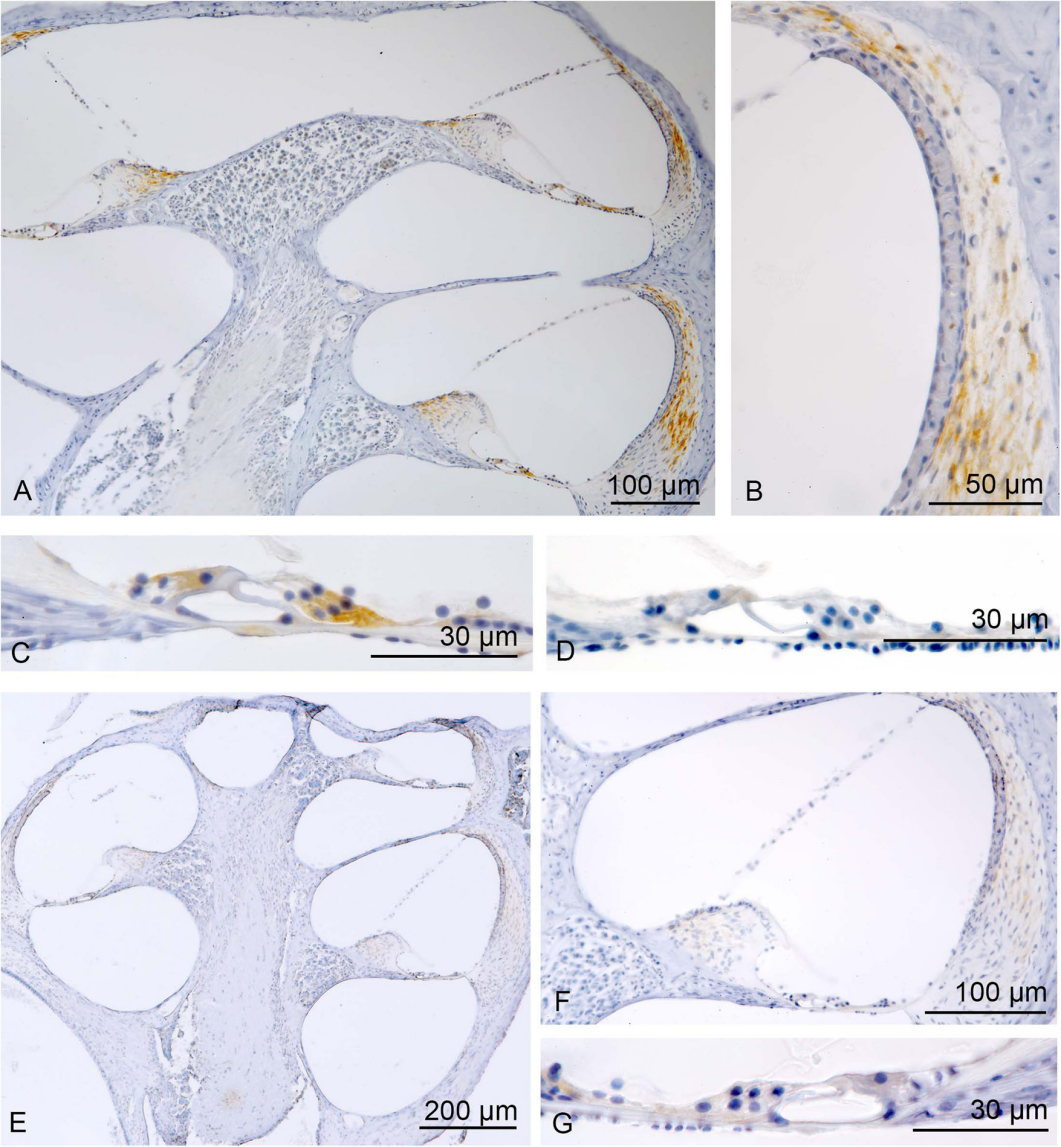
**Cleaved caspase-3.** Sepsis mice with a high hearing loss showed positive CC-3 cells mainly in the lateral wall and in the organ of Corti (A,C). In the lateral wall, positive immunostaining was observed in type I and type V fibrocytes (A). In the organ of Corti the Deiters’ cells and inner hair cells were strongly stained, while no reactivity was found in the cell body of the outer hair cells (C). In sepsis mice with a low hearing loss altogether fewer cells were stained and immunoreactivity intensity was lower (B,D). In the sham mice only a faint staining of some fibrocytes of the spiral ligament was found (E-G).

As shown in Fig. 2, sepsis mice with a high hearing loss showed positive CC-3 cells mainly in the lateral wall and in the organ of Corti. In the lateral wall, positive immunostaining was observed in type I and type V fibrocytes. In the organ of Corti the Deiters’ cells and inner hair cells were strongly stained, while no reactivity was found in the cell body of the outer hair cells. Spiral ganglion neurons revealed only modest immunoreactivity. There was also a discrete labelling of the fibrocytes of the spiral limbus.

In sepsis mice with a low hearing loss, altogether fewer cells were stained and immunoreactivity intensity was lower. While in the lateral wall fewer fibrocytes were stained, in the inner hair cells no reactivity was found.

In the sham mice only a faint staining of some fibrocytes of the spiral ligament was found.

The vestibular organ was devoid of any immunoreactivity. The hair cells, sensory epithelium and accessory structures of the vestibular organ were configured regularly (Fig. 2).

### BCL-2

In the sepsis mice with high hearing loss we found a staining of BCL-2 in the spiral ganglion cells. A higher immunoreactivity was found especially in clusters of neurons that regularly appear preferentially in the apical turn of C57BL mice. In the organ of Corti the inner hair cells and the Deiters’ cells showed an intense staining and a moderate labelling in the outer hair cells was found. BCL-2 staining was observed also in the outer sulcus epithelial cells.

In the sepsis mice with low hearing loss the staining intensity of BCL-2 was nearly the same as in the mice with high hearing loss.

In the sham mice lower staining reactivity in the spiral ganglion cells and lateral wall was observed. In the organ of Corti the reactivity was comparable to the sepsis mice.

**Fig. 3.**
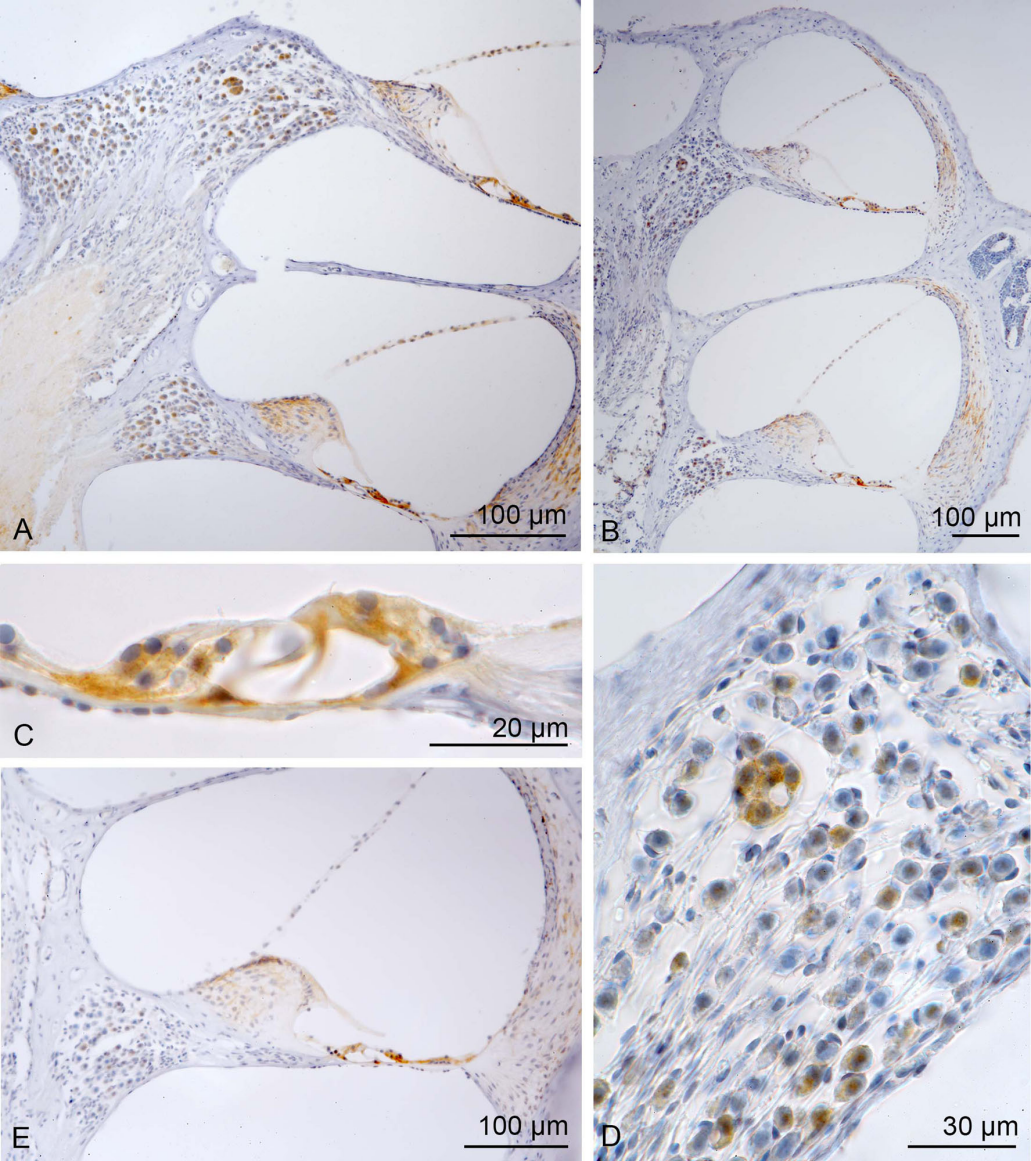
**Immunostaining of the antibody BCL 2.** BCL2 showed nearly the same staining intensity in sepsis mice with high hearing loss (A) and low hearing loss (B). The inner hair cells (C), Deiters’ cells (C) and spiral ganglion cells, especially clusters of neurons (D), which are preferentially in the apical turn, showed a positive immunoreactivity. In the sham mice lower staining reactivity in the spiral ganglion cells and lateral wall was found (E).

Also in the vestibular system a moderate staining of BCL-2 was observed in sepsis mice in the utricle (Fig. 3).

### BAX

**Fig. 4.**
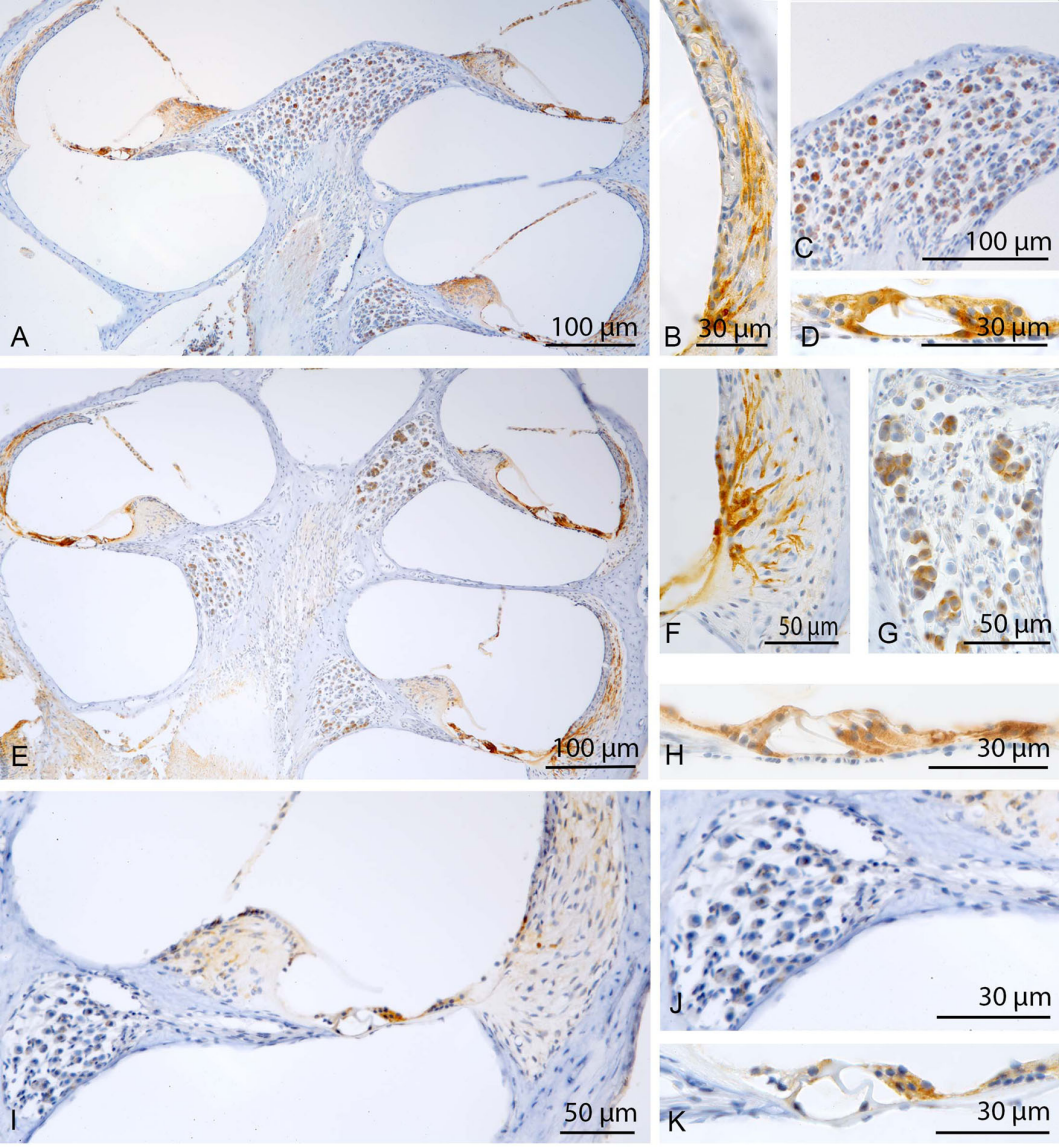
**Immunostaining of the antibody BAX.** BAX is distributed in sepsis mice with high hearing loss (A-D) and in sepsis mice with low hearing loss (E-H) mainly in type I, II and V fibrocytes (A,B,E), root cells (B,F), inner and outer hair cells, supporting cells (D,H) and in the Reissner’s membrane (A,E). Additionally, in high hearing loss a positive immunostaining was found in the intermediate cells in the stria vascularis (B). In the sham mice only slight staining in the inner hair cells and Deiter′s cells was found (I-K).

In sepsis mice with high hearing loss BAX was distributed mainly in type I, II, and V fibrocytes, root cells, outer and inner hair cells and supporting cells were strongly stained. Additionally a positive immunostaining was observed in the spiral ganglion cells, intermediate cells in the stria vascularis, the Reissner’s membrane and in the outer sulcus epithelial cells. Only a faint staining in the vestibular system was observed (Fig. 4).

In sepsis mice with low hearing loss a positive immunostaining was observed in the spiral ganglion cells, Reissner’s membrane, type I fibrocytes, root cells, Deiters’ cells and moderately in the inner hair cells. The outer hair cell showed a marginal staining. No staining in the stria vascularis was observed.

In the sepsis mice discrete labelling of BAX in the utricle and ampulla was found. In the utricle, especially in the type II, supporting cells and some type I cells, a positive immunoreactivity was found. Additionally, in the ampulla the central type I cells showed an immunoreactivity, while in the peripheral area of the ampulla only supporting cells were labelled.

In the sham mice only slight staining in the inner hair cells and Deiter's cells was found, while the ganglion cells, lateral wall and outer hair cells did not show any staining (Fig. 4).

Table 2 shows the mean percentage of positive immunoreactive area in the spiral ligament in sepsis mice with high and low hearing loss and in sham mice. In sepsis mice with high hearing loss a higher reactivity of BAX (14.96) than in sepsis mice with low hearing loss (11.94) was shown, while only a moderate staining was observed in sham mice (4.91).

**Table 2.**
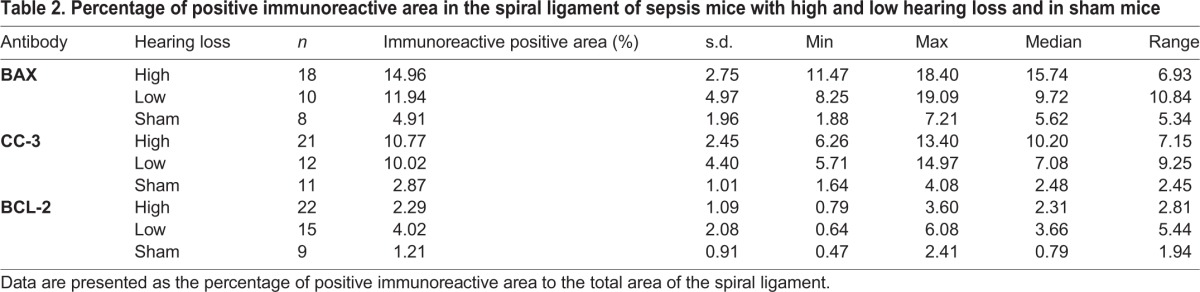
**Percentage of positive immunoreactive area in the spiral ligament of sepsis mice with high and low hearing loss and in sham mice**

CC-3 showed nearly the same mean percentage of positive immunoreactive area in sepsis mice with high hearing loss (10.77) and in low hearing loss (10.09), but only a slight reactivity in sham mice (2.87).

The staining of BCL-2 in the spiral ligament was highest in sepsis mice with low hearing loss (4.02). A lower reactivity in sepsis mice with high hearing loss (2.29) and only a minimal reactivity in the sham mice (1.21) was observed.

**Fig. 5.**
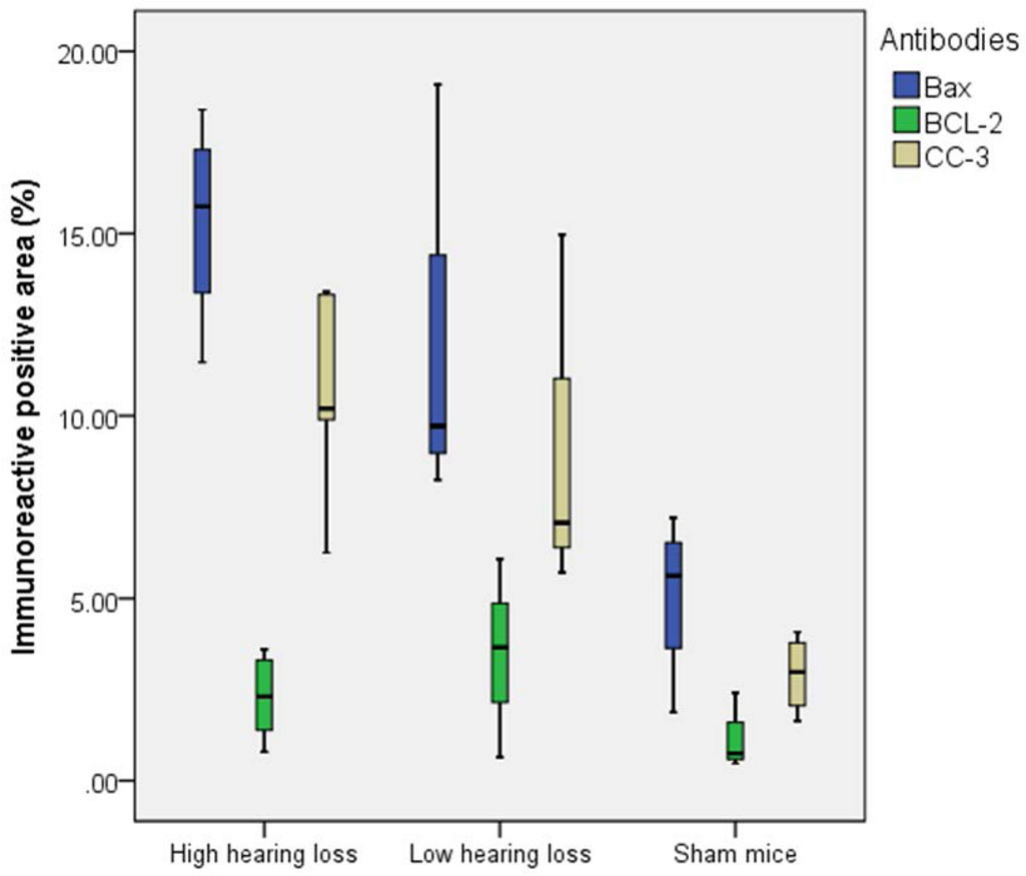
**Percentage of the immunostained positive area.** The percentage of the immunostained positive area in the spiral ligament in sepsis mice with high hearing loss, sepsis mice with low hearing loss and in sham mice are shown. In sepsis mice a higher activity of BAX (*P*=0.027) and CC-3 (*P*=0.024) can be observed. BCL-2 showed no statistically significant alterations. Box and whiskers indicate interquartile range and minimum/maximum, respectively.

The percentage of the immunostained positive area in the spiral ligament showed a significant correlation with the grade of hearing loss for BAX (*P*=0.027) and CC-3 (*P*=0.024), but no statistically significant correlation could be found for Bcl2 (*P*=0.222) (Fig. 5).

## DISCUSSION

A previous publication has demonstrated that experimental sepsis can lead to a significant hearing impairment in mortally sick mice. In this murine model an apoptosis in the supporting cells of the organ of Corti and a disturbance of the synapses of the inner hair cells could be described (Schmutzhard et al., 2013). A critical point in this study was when a severe sepsis was induced and the auditory evoked brainstem responses were measured at the peak of the disease; these animals had already reached clinical parameters which indicate a moribund state with a lethal outcome. Hankenson et al. identified weight loss of 0.05 g daily and a non-transient decreased body temperature of 34.5°C as parameters in murine HSV infection which would not be survived (Hankenson et al., 2013).

Therefore, the question arises if the observed functional and morphologic impairment of the inner ear develops in animals surviving sepsis.

In the current study experimental sepsis has been induced with cecal ligation puncture. Rittirsch et al. defined two grades of sepsis in CLP technique: a ligation of the cecum at the middle containing 50% of the cecum results in a survival rate of 40%, this has been referred to as a mid-grade sepsis. In contrast, if 75% of the cecum is ligated a mortality of 100% can be expected and has been defined as high-grade sepsis (Rittirsch et al., 2009). In this study a mid-grade sepsis with a mortality of 60% was intended; therefore a medium cecal ligation, as described by Rittirsch et al. (2009), was performed. In this investigation we found a survival rate of 40% confirming the results of Rittirsch et al. (2009) and the quality of the experiment.

In the initial objective hearing test a nearly similar mean hearing threshold at an average of 41.5 dB in all three groups could be observed, which is considered a regular hearing threshold for C57BL/6 mice (Zheng et al., 1999).

All surviving mice were scheduled for a second ABR measurement on day 7. In the surviving sepsis mice the mean hearing threshold increased significantly for 9.8 dB. In contrast the hearing thresholds of the sham and control group remained stable with a mean difference of only ±0.2 dB. These results confirm prior findings, describing an average hearing loss of 8.1 dB in lethal septic mice and no change in the control and sham group (Schmutzhard et al., 2013).

In a malaria mouse model an apoptosis in the fibrocytes of the spiral ligament and a breakdown of the blood-labyrinth barriers as pathological mechanisms of the hearing loss could be observed (Schmutzhard et al., 2012). In a prospective multicentre cohort study Schmutzhard et al. investigated otoacoustic emissions in children with severe malaria and found a significant impairment of transitory otoacoustic emissions. These findings confirm the involvement of the inner ear in malaria (Schmutzhard et al., 2015). In this study we controlled these findings in a non-lethal sepsis model and investigated the activity of the apoptosis markers in the spiral ligament.

The histomorphologic work-up for the surviving mice revealed a good structure preservation. Furthermore the immunolabelling for the pro-apoptotic markers BAX and CC-3 was positive in the Deiter’s cells and inner hair cells as well as in the spiral ligament. For this the percentage of positive immunoreactive area in the spiral ligament was compared in mice with and without sepsis. A significant activity of BAX (*P*=0.027) and CC-3 (*P*=0.024) in sepsis mice with a high hearing loss could be found. Animals of the sham group did not show any reaction or only a very mild staining in the supporting cells of the cochlea.

A Bax/Bcl-2 ration favouring Bax expression and an additional caspase-3 upregulation is a secure sign for induced apoptosis (Salakou et al., 2007), so these findings show that a mild sepsis in mice can lead to a hearing impairment due to apoptosis of supporting cells of the organ of Corti, as well as in the lateral wall, especially in the type I fibrocytes of the spiral ligament and changes in the inner hair cells.

A positive CC-3 staining in the Deiter’s cells has been described previously in lethal sepsis mice (Schmutzhard et al., 2013). A new histopathologic alteration in sepsis mice is the positive reaction of the spiral ligament especially in the area of type I and type V fibrocytes. The type I fibrocytes, which are located between the stria vascularis and the bony wall of the cochlea and the type V fibrocytes, which are located in the suprastrial area between the scala vestibule and the cochlear wall, are essential for the maintenance of ionic and metabolic homeostasis in the inner ear and many other functions. Type V fibrocytes pump K+ from the perilymph and produce a K+ flow to Type I fibrocytes. Type I fibrocytes are electrically connected to the cells in the stria vascularis. A dysfunction of these fibrocytes leads to a disturbance of the endocochlear potential (Jagger and Forge, 2015; Raphael and Altschuler, 2003).

The antiapoptotic marker BCL-2 showed a slight upregulation in the sepsis mice with low hearing loss, although also in the sham mice a basal expression level could be observed. These findings suggest that the antiapoptotic potency of BCL-2 protects against hearing loss.

The pathophysiology is unknown, but several studies showed that the signalling cascades of TNF are involved in hearing impairment. TNF-α and TNF receptor interactions have a central role in the pathogenesis of the inflammatory response, and cause programmed cell death and cell proliferation (Uchida et al., 2014).

Baratz et al. proved the importance of TNF-α in neuroinflammation and brain damage. They found that a TNF-α synthesis inhibitor may mitigate secondary brain damage after a traumatic brain injury (Baratz et al., 2015; Tweedie et al., 2012).

However, recent research on the pathophysiologic mechanisms underlying sepsis indicates that sepsis is a complex and dynamic disease process involving excessive and suppressed inflammatory and immune responses (Schulte et al., 2013). Inflammatory response is a balanced response between pro-inflammatory mediators (systemic inflammatory response, SIRS) and anti-inflammatory mediators (compensatory anti-inflammatory response, CARS). SIRS mediators such as TNF-α and interleukin-1 (IL-1), IL-6 and IL-12 activate the immune-inflammatory system, which can then be de-activated through the expression of CARS mediators, including IL-1 receptor antagonist, TGF-ß, IL-4, IL-10 and IL-13. In a septic shock the balance of SIRS and CARS is disturbed, resulting in an exaggerated and dysfunctional inflammatory response, which is associated with apoptosis induction (Buras et al., 2005).

This suggests that a systemic inflammation induces an inflammatory pathway in the inner ear which causes a secondary damage of inner ear structures resulting in hearing impairment. Therefore, a prophylactic local anti-inflammatory therapy – like a TNF-α-neutralizing agent – should be discussed in a systemic inflammation to prevent the sensorineural hearing loss. This has been shown to be effective by Aminpour et al. (2005) in hearing loss due to experimental induced pneumococcal meningitis.

Further investigations in this direction could lead to interesting findings and could create new therapeutic options.

### Conclusion

This finding also supports the hypothesis that a mild sepsis leads to a significant hearing impairment due to apoptosis of supporting cells of the organ of Corti and changes in the inner hair cells and spiral ligament. In sepsis mice the mean hearing thresholds increased for 9.8 dB, while the thresholds in the control mice remained stable. To prevent this severe life quality restricting disease a prophylactic therapy is necessary and should be a focus of further research.

## MATERIAL AND METHODS

The animal studies conformed to the Austrian guidelines for the care and use of laboratory animals and were approved by the Austrian Ministry Science with the reference number BGBI.I Nr. 114/2012.

### Basic settings and study design

In this study two-month-old C57BL/6 mice (Charles River, Sulzfeld, Germany) were used. An initial objective hearing test was performed with ABR. Animals with thresholds below 40 dB at 16 kHz were eligible. Sepsis was induced in 35 mice with cecal ligation puncture; 15 mice underwent sham surgery (laparotomy, no ligation, no puncture) and 13 mice served as control (identical housing and treatment, but no intervention). The course of sepsis was monitored for signs of prostration, lowered temperature and weight loss at 12-hour intervals. On day 7 in all surviving mice a second ABR measurement was performed. Deeply anesthetised the animals were sacrificed afterwards. Within 7 min after death the inner ears were prepared and fixed for further morphologic and immunohistochemical examination.

### Cecal ligation puncture mouse model

The mice were deeply anesthetised with intraperitoneal ketamine hydrochloride (Graeub^®^, Senden-Bösensell, Germany) (67.5 mg/kg body weight), xylazine hydrochloride (Bayer^®^, Leverkusen, Germany) (5.4 mg/kg body weight) and atropine sulfate (Nycomed^®^, Linz, Austria) (0.085 mg/kg). After immobilizing the animal, the fur of the belly was trimmed and disinfected with 90% alcohol. A median incision was done on the linea alba. The cecum was mobilized and ligated with a 4.0 Vicryl suture (Ethicon, Norderstedt, Germany) in the middle of the cecum in order to induce an anticipated mortality of 60% (Rittirsch et al., 2009). The perforation, saving the blood vessels, was done with a 21 gauge needle, exposing small droplets of faeces on both perforation sides. The cecum was repositioned and the incision closed in layers with 4.0 Vicryl sutures. Afterwards, the animals were rescued with a subcutaneous injection of 37°C warm sodium chloride (1 ml/20 g body weight). After the procedure, the animals were kept on a 37°C warm plate with free access to unlimited food and water.

### Methods of ABR recording

The animals were anaesthetised by intraperitoneal injection of ketamine hydrochloride (67.5 mg/kg body weight), xylazine hydrochloride (5.4 mg/kg body weight) and atropine sulphate (0.085 mg/kg). Mice were kept on a pre-warmed (37.0°C) gel pad, which provides a constant body temperature during measurements.

Auditory nerve responds were measured with a custom made portable system controlled by the software Audiology Lab (Marcus Müller, University Tübingen, Germany) in a custom-made electrically shielded sound attenuated chamber. Potentials were recorded via three subcutaneous needle-electrodes. Tone bursts of 4, 8, 16 and 32 kHz were used as stimuli. Tone bursts were produced by two different transducers. Starting with 0 dB the stimuli increased by 5 dB up to 90 dB. Hearing thresholds were determined as the minimum stimulation level that produced a clearly recognizable potential. After amplification and band pass filtering (100 dB, 0.3–5 kHz), electrical signals were averaged over 128 repetitions of stimulus pairs. The ABR results were evaluated by two independent Otologists in a blinded manner.

### Specimen preparation

Immediately after sacrifice, the cochleae were harvested and dissected within 7 min. The round and the oval windows were opened and 4% formaldehyde in phosphate buffered saline (PBS), pH 7.4, freshly prepared from paraformaldehyde was perfused into the fluid spaces. Subsequently inner ears were immersed in the fixative. Decalcification was performed with an 8-hour exposure of tissue in 20% EDTA (Titriplex III; Merck, Darmstadt, Germany) in PBS, pH 7.4, at 37°C. For immunohistochemical examination, cryoembedded serial sections (10 μm) were cut on a cryostat (LEICA CM 3050), mounted on glass slides (SuperFrost^®^Plus, Menzel-Gläser, Braunschweig, Germany), air-dried for 1 h and stored at −20°C.

### Immunostaining

Slides were immunostained with an automated system (Ventana Roche Discovery Classic) according to the DAB-MAP discovery research standard procedure to guarantee reproducible results. Slides were rehydrated in distilled water and exposed to primary antibodies without any antigen unmasking strategies. Antibodies raised against BAX (1:50, polyclonal rabbit sc-529, Santa Cruz Biotechnology Inc. mapping to 7B5 mouse locus), Cleaved caspase-3 (1:400x, polyclonal rabbit, Cell Signalling Technology, Asp175, recognizes the large fragment -17/18kDa-of activated caspase 3, Danvers, MA, USA) and BCL-2 (1:200, polyclonal rabbit sc-492, Santa Cruz Biotechnology Inc.), raised against a peptide mapping at the N-terminus of human Bcl-2 were incubated for 60 min and detected with biotinylated secondary antibodies (1:400, biotinylated donkey anti-rabbit, 711-065-152, Jackson ImmunoResearch, PA, USA, incubation 30 min) visualized by a peroxidase-antiperoxidase system developed with Diaminobenzidin intensified with copper sulfate. Blocking of endogenous peroxidase was accomplished by competitive blocking with an excess of peroxidase. For control purposes, representative sections were processed in the same way by substituting primary antibodies with isotype matching immunoglobulins (1:200, rabbit polyclonal IgG, Abcam,ab27478). Slides were counterstained with haematoxylin, dehydrated in ascending grades of isopropanol cleared in xylene and mounted with Entellan^®^ (Merck^®^, Germany).

Antibodies utilised in the study for immunohistochemical analyses are summarized in Table S1.

### Imaging

The immunohistochemical slides were digitised and analysed with TissueFAXS (TissueFAXS Plus – Cell Analysis System, TissueGnostics^®^ GmbH, Vienna, Austria) and evaluated with the analysis software HistoQuest (TissueGnostiscs GmbH, Vienna, Austria). The system consisted of a Zeiss Imager Z2 microscope equipped with a 3 megapixel area scan colour camera Pixelink PL-623 CF. The motorised stage from Maerzhauser had an 8-slice insert easing the batch scan. The white light was delivered by a VISLED lamp based on LED technology which ensured a stable reproducible colour temperature over the entire light intensity spectrum. The image acquisition phase was done with a Zeiss 20x, 0.6 apochromat objective. Every scanning project acquired a non-saturated white image for shading compensation. The sections were investigated and the spiral ligament was manually selected and marked for analysis using regions of interest (ROI). The computer analysis was done using HistoQuest 3.5 cytometry software calculating the mean optical density of the immunohistochemical signal across the total ROI area. The software splits the colour into marker-specific channels using an integrated colour separation method termed single reference shade. This approach can reliably separate Haematoxylin and colorimetric DAB developed antibody immunoreactivity. A manual background threshold was set once interactively to extract positive areas and used throughout all slides processed.

### Statistical analysis

ABR measurements were performed in each animal before treatment at the start and after the treatment at the end of the observation period. Pure tone average (PTA) was calculated as the mean of the left and right ear ABR-thresholds for the frequencies 4, 8, 16 and 32 kHz for each animal. For descriptive statistics whisker plots were used to demonstrate the cumulative PTA score. To test treatment effects (CLP versus sham or control), one-way analysis of covariance was used with post-treatment PTA as the outcome variable adjusted for pre-treatment PTA as a covariate, with a *P* value less than 0.05 considered significant.

The measurement of the immunohistochemical slides were exported from the validated analysed projects to Microsoft^®^ Excel^®^ containing all relevant data: total analysed area, optical density/µm² and segment positive area µm² of each domain.

Statistical analysis was performed in SPSS 21 (SPSS Statistics 21, IBM, Armonk, USA) using the data from exported Microsoft^®^ Excel^®^.
