# CORRECTION: Surviving murine experimental sepsis affects the function and morphology of the inner ear

**DOI:** 10.1242/bio.028761

**Published:** 2017-09-15

**Authors:** Natalie Fischer, Nina Maria Mathonia, Georges Hollerich, Julian Veser, Leyla Pinggera, Daniel Dejaco, Rudolf Glueckert, Anneliese Schrott-Fischer, Peter Lackner, Herbert Riechelmann, Joachim Schmutzhard

There was an error published in *Biology Open* **6**, 732-740; doi: 10.1242/bio.024588

These errors are detailed below and the original article has been changed correspondingly.

The name of the co-author, Georges Hoellerich, was spelt incorrectly and has been changed to Georges Hollerich.

